# Effects of Fullerenols on Mouse Brain Microvascular Endothelial Cells

**DOI:** 10.3390/ijms18081783

**Published:** 2017-08-17

**Authors:** Michael K. Schuhmann, Felix Fluri

**Affiliations:** Department of Neurology, University of Würzburg, 97080 Würzburg, Germany; schuhmann_m@ukw.de

**Keywords:** fullerenes, blood-brain barrier, mouse brain microvascular endothelial cell culture, inflammation, tight junctions, adhesion molecules

## Abstract

Fullerenols, water-soluble C60-fullerene derivatives, have been shown to exert neuroprotective effects in vitro and in vivo, most likely due to their capability to scavenge free radicals. However, little is known about the effects of fullerenols on the blood–brain barrier (BBB), especially on cerebral endothelial cells under inflammatory conditions. Here, we investigated whether the treatment of primary mouse brain microvascular endothelial cells with fullerenols impacts basal and inflammatory blood–brain barrier (BBB) properties in vitro. While fullerenols (1, 10, and 100 µg/mL) did not change transendothelial electrical resistance under basal and inflammatory conditions, 100 µg/mL of fullerenol significantly reduced erk1/2 activation and resulted in an activation of NFκB in an inflammatory milieu. Our findings suggest that fullerenols might counteract oxidative stress via the erk1/2 and NFκB pathways, and thus are able to protect microvascular endothelial cells under inflammatory conditions.

## 1. Introduction

Multiple sclerosis (MS) is a chronic inflammatory, progressive and degenerative disease of the central nervous system (CNS). Histopathologically, MS is characterized by demyelinated lesions, immune cell infiltration, axonal injury, and astrogliosis [1]. The migration of peripheral immune cells through an impaired blood–brain barrier (BBB) into the cerebral parenchyma is an early and pivotal event in the pathogenesis of this disease [2]. The BBB is formed by perivascular astrocytes, pericytes, and highly specialized endothelial cells that are connected by tight junctions (TJ) such as occludin and claudins, thereby reducing the translocation of immune cells from the blood into the cerebral tissue. Regarding MS, however, activated monocytes synthesize a range of pro-inflammatory factors, such as tumor necrosis factor-α (TNF-α) and reactive oxygen species (ROS), e.g., nitric oxide, which affects the integrity of the BBB and allows the enhanced adhesion and migration of leukocytes. Particularly, TNF-α has been demonstrated to promote transendothelial leukocyte migration by upregulation of vascular adhesion molecules such as intercellular adhesion molecule 1 (ICAM-1) and vascular cell adhesion molecule 1 (VCAM-1) [3,4]. Furthermore, TNF-α induces the production of ROS by brain endothelial cells, which further injures the integrity of the BBB [5,6]. Altogether, there is increasing evidence that oxidative stress plays a crucial role in the pathogenesis of MS. ROS are also generated excessively by activated microglia and have been implicated as mediators of demyelination and axonal damage in MS [7]. In addition, redox reactions are implicated in the activity of matrix metalloproteinases (MMPs), which are important for T-cell trafficking into the cerebral tissue [8]. ROS can also activate several transcription factors that upregulate the expression of genes involved in human MS, such as erk1/2 [9] or NFκB [10]. Moreover, the direct examination of MS plaques revealed an increase in free radical activity and decreased levels of relevant antioxidants such as glutathione (GSH) [11]. When taken together, the aforementioned data indicate that antioxidants might be a promising therapeutic strategy for restoring the BBB in MS patients. Agents that have shown promising antioxidative properties are fullerenes. Fullerenes are carbon-made and sphere-shaped, and can consist of a varying number of sp2 hybridized carbon atoms [12,13]. Such peculiar chemistry on the carbon cage confers specific features to fullerenes, such as radical scavenger and redox properties [12,13]. Based on their high affinity for free radicals, fullerenes have been described as neuroprotective in cortical cell cultures [14]. A single C_60_ sphere is capable of catching as many as 34 radicals [15]. Fullerene derivatives also cause the recovery of GSH [16] and nitric oxide (NO) [17], thus supporting radical removal. In addition, fullerene derivatives have been shown to bear neuroprotective properties in in vivo models of ischemic stroke, as well as in a mouse model of progressive MS [18,19,20]. However, even though circulating fullerenes are in direct contact with vascular endothelial cells, few studies have addressed the effect of fullerenes on cerebral endothelial cells [21,22]. In particular, the capacity of fullerenes to modulate the BBB properties of brain-derived microvascular endothelial cells has not been investigated so far. With these considerations in mind, we investigated whether hydroxylated fullerenes i.e., fullerenols (1) protect the integrity of the BBB, using transendothelial electrical resistance (TEER) as a surrogate marker and the expression of occludin, claudin 3 and claudin 5; (2) counteract the expression of vascular adhesion molecules (VCAM-1 and ICAM-1); and (3) impact the erk1/2 or NFκB p65-signaling pathway.

## 2. Results

### 2.1. Fullerenols Modulate Glutathione (GSH) Content in Inflamed MBMEC

**Figure 1:**
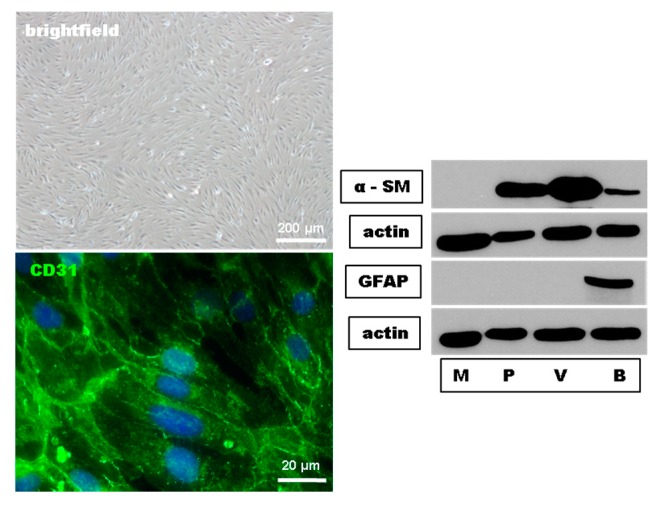
Histological characterization and Western blot purity control of mouse brain microvascular endothelial cells (MBMEC). Phase contrast image of confluent MBMEC revealed that the cells express a spindle-shaped morphology and a constant monolayer. Micrographs of immunofluorescence staining against Hoechst (blue) and CD31 (green). Western blot analysis of the expression of alpha smooth muscle actin (α-SM) and Glial Fibrillary Acidic Protein (GFAP) in MBMEC (M) compared to pericytes (P), mouse brain microvessels (V), and mouse brain lysate (B). Actin was used as loading control.

In a first set of experiments, we proofed the purity and the endothelial phenotypic morphology of our primary mouse brain-derived microvascular endothelial cell (MBMEC) culture using phase-contrast microscopy, immunohistochemistry, and Western blot analyses. MBMEC expressed a spindle-shaped morphology and the endothelial cell marker CD31. Using Western blot analyses, the absence of the pericyte marker α smooth muscle actin (α-SM) and the astrocyte marker glial fibrillary acidic protein (GFAP) confirmed the purity of the MBMEC cultures (Figure 1).

**Figure 2:**
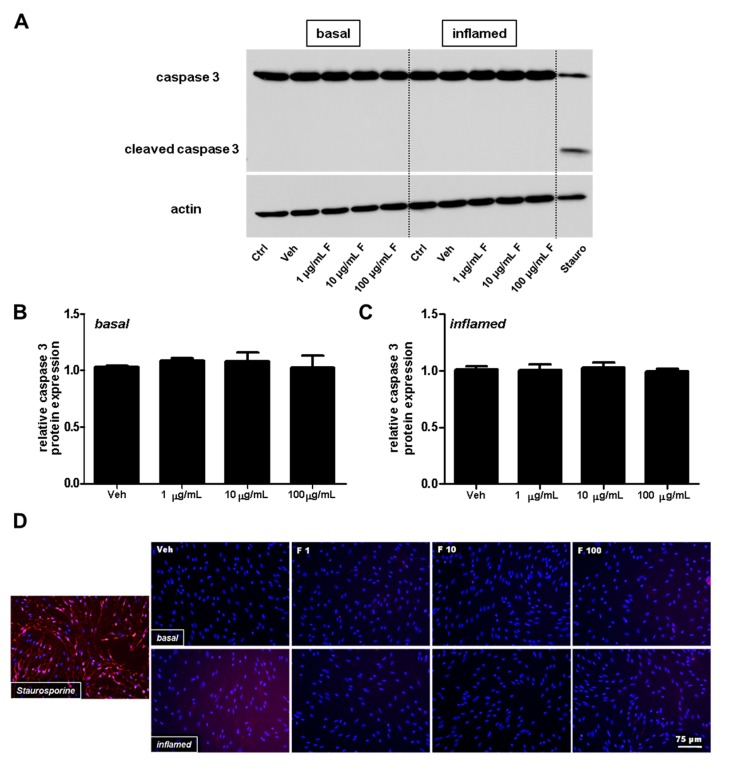
Analysis of apoptosis in mouse brain microvascular endothelial cells (MBMEC) exposed to different doses of fullerenols under basal and inflammatory conditions. (**A**) Representative Western blot analyses of full-length caspase 3 protein expression in MBMEC cultures after exposure to interferon-γ and tumor necrosis factor-α (I + T; 100 IU each) and treatment with fullerenols (F; 1, 10 and 100 µg/mL; *n* = 3) for 18 h compared to cultures under basal conditions. Staurosporine treatment (Stauro; 1 μM for 2 h) was used as a positive control; (**B**) Densitometric quantification of the amount of full-length caspase 3 in MBMEC, treated with different doses of fullerenols or vehicle (normalized to untreated control cells) cultured for 18 h under basal conditions; (**C**) Densitometric quantification of the amount of full-length caspase 3 in MBMEC, treated with different doses of fullerenols or vehicle (normalized to untreated control cells) cultured for 18 h under inflammatory conditions; (**D**) Representative immunofluorescence images of cleaved caspase 3 (red) counterstained with Hoechst (blue) in MBMEC treated with fullerenols (F 1, 1 µg/mL; F 10, 10 µg/mL; F 100, 100 µg/mL) for 18 h under basal or inflamed (I + T; 100 IU each) conditions. Staurosporine treatment (1 μM for 2 h) was used as a positive control. Scale bar counts for all immunofluorescence images. Ctrl, untreated control; Veh, vehicle treatment.

To exclude changes of MBMEC barrier properties due to apoptosis, we next investigated the caspase 3 protein amount using Western blot analysis, followed by densitometric quantification. The findings were further verified by immunofluorescence analysis of cleaved caspase 3. Staurosporine, a frequently used agent to induce Ca^2+^-dependent cell death pathways, served as a positive control for our apoptosis assays, and clearly induced cleavage of caspase 3. Under physiological and inflammatory conditions (interferon (IFN)-γ and TNF-α), fullerenol (i.e., doses of 1, 10 and 100 µg/mL) neither decrased the full-length caspase 3 (Figure 2A–C) nor induced an increase in cleaved caspase 3 expression (Figure 2D).

**Figure 3:**
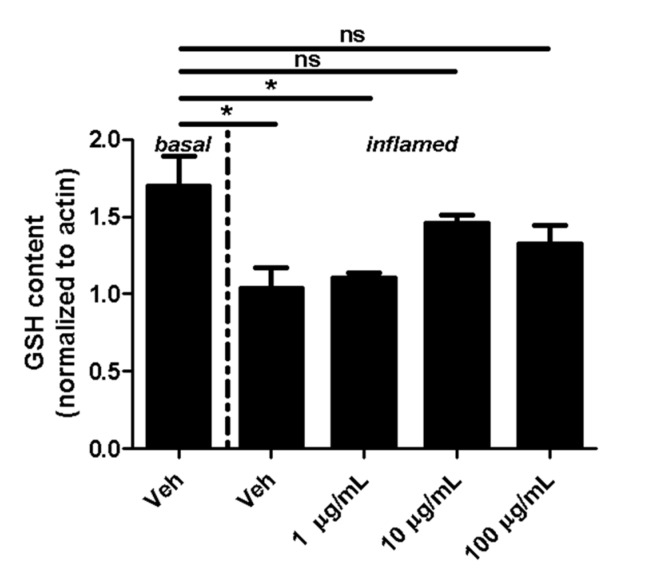
Effect of fullerenols on the glutathione (GSH) content. Quantification of GSH content (normalized to β-Actin; assessed by densitometric quantification of Western blot band intensity) in MBMEC after exposure to interferon-γ and tumor necrosis factor-α (inflamed; I + T; 100 IU each) and treatment with fullerenol (F; 1, 10 and 100 µg/mL; *n* = 3) for 18 h compared to cultures under basal (homeostatic milieu) conditions . Veh, vehicle treatment. * *p* < 0.05; ns, not significant.

Fullerene derivatives have been suggested to modulate the expression of GSH, which is an important cellular antioxidant [16]. Therefore, we next investigated whether fullerenols alter the GSH content of MBMEC cultured for 18 h under basal or inflammatory conditions. Inflammation clearly decreased the content of cellular GSH in MBMEC treated with vehicle (Veh basal: 1.7 ± 0.2; Veh inflamed: 1.0 ± 0.2; *p* < 0.05). Fullerenol in the lowest dose did not contribute to the recovery of GSH (1.1 ± 0.03; *p* < 0.05). In contrast, when fullerenol was added in higher doses to the cell cultures, the GSH-content of MBMEC reached almost the same values as when measured under basal conditions (Figure 3).

### 2.2. Fullerenols Do Not Modulate MBMEC Barrier Functions

**Figure 4:**
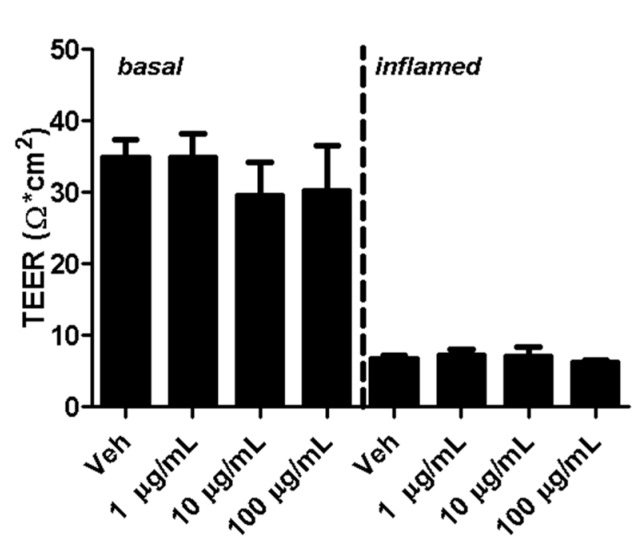
Transendothelial electrical resistance (TEER) of mouse brain microvascular endothelial cell (MBMEC) cultures. TEER values of MBMECs 18 h after exposure to I + T (100 IU each; inflamed) and treatment with fullerenols (1, 10 and 100 µg/mL; *n* = 3) compared to cultures under basal conditions. Veh, vehicle treatment.

Transendothelial electrical resistance (TEER) is a widely accepted indicator for the integrity of cellular barriers [23]. Five days after seeding MBMECs, fullerenols were added to cell cultures under basal and inflamed conditions. Analysis of TEER 18 h after MBMEC treatment revealed a clear decrease in cells exposed to IFN-γ and TNF-α. Adding fullerenol to the cultures did not impact TEER compared to untreated controls under basal or inflammatory conditions (Figure 4).

**Figure 5:**
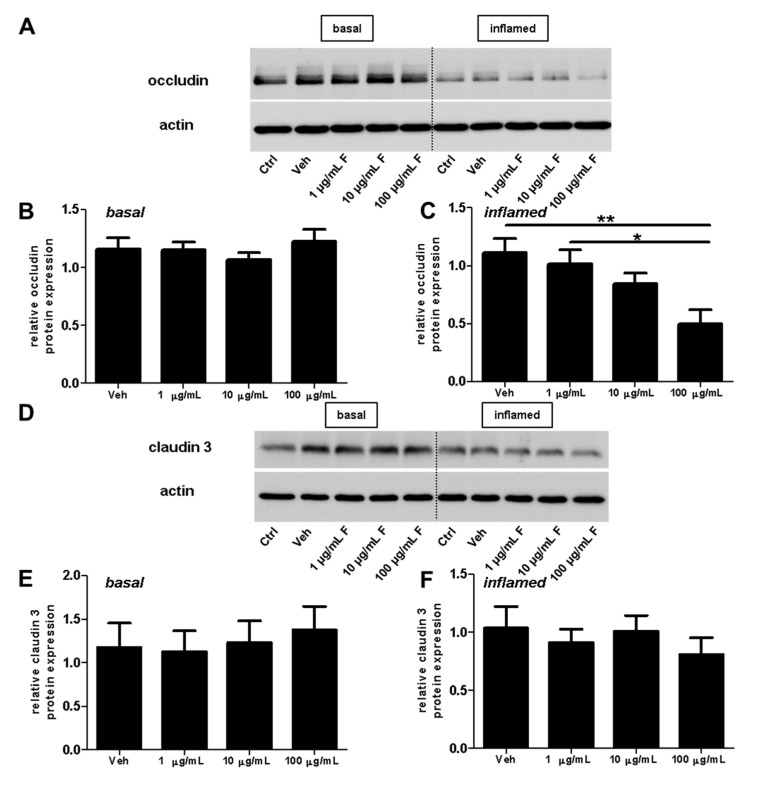

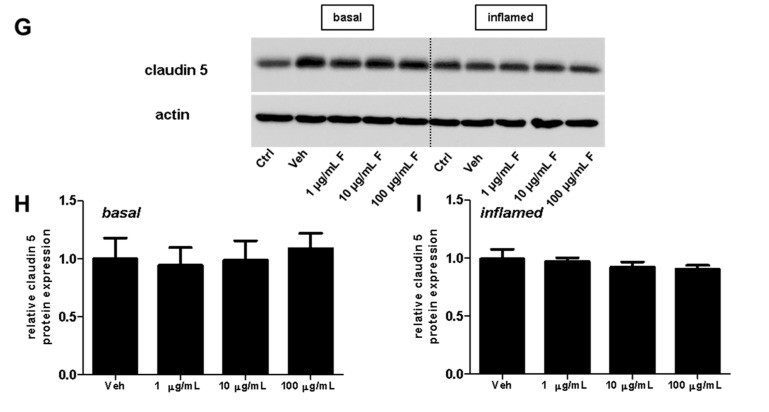
Effect of fullerenols on the amount of junctional proteins in mouse brain microvascular endothelial cell (MBMEC) cultures under basal and inflammatory conditions. Western blot analysis and densitometric quantification (normalized to basal or inflamed untreated control cell cultures) of the amount of occludin (**A**–**C**), claudin 3 (**D**–**F**) and claudin 5 (**G**–**I**) proteins in MBMEC after exposure to interferon-γ and tumor necrosis factor-α (I + T; 100 IU each) and treatment with fullerenol (F; 1, 10 and 100 µg/mL; *n* = 4–5) for 18 h (**C**,**F**,**I**) compared to cultures under basal (homeostatic milieu) conditions (**B**,**E**,**H**). β-Actin was used as loading control. Ctrl, untreated control; Veh, vehicle treatment. * *p* < 0.05; ** *p* < 0.01.

**Figure 6:**
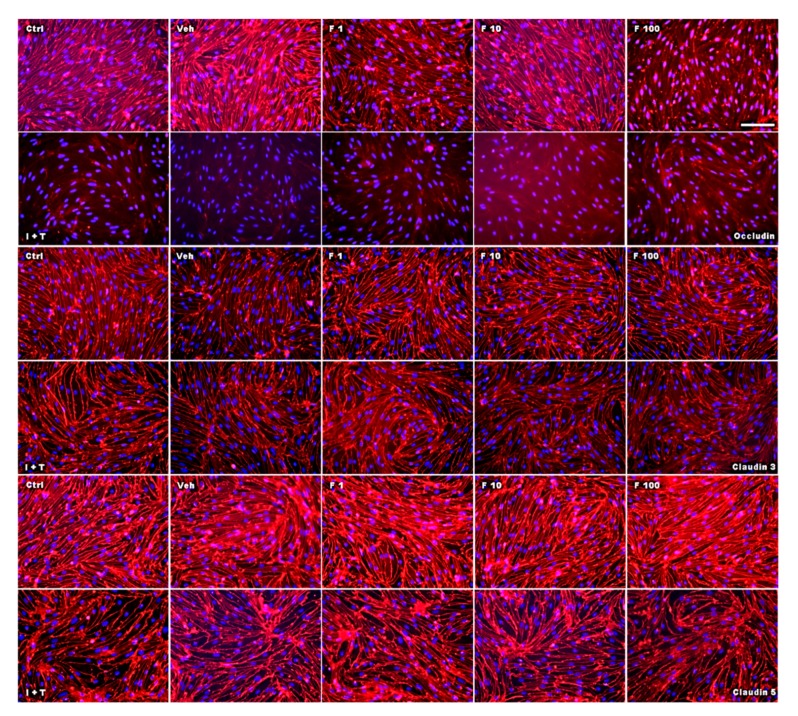
Effect of fullerenol on the distribution of the junctional proteins in mouse brain microvascular endothelial cell (MBMEC) cultures under basal and inflammatory conditions. Immunofluorescence images of tight junction molecules (red) counterstained with Hoechst (blue) in MBMEC treated with fullerenols (F 1, 1 µg/mL; F 10, 10 µg/mL; F 100, 100 µg/mL) for 18 h under basal or inflammatory (I + T; 100 IU each) conditions. Scale bar (100 µm) counts for all immunofluorescence images. Ctrl, untreated control; Veh, vehicle treatment.

**Figure 7:**
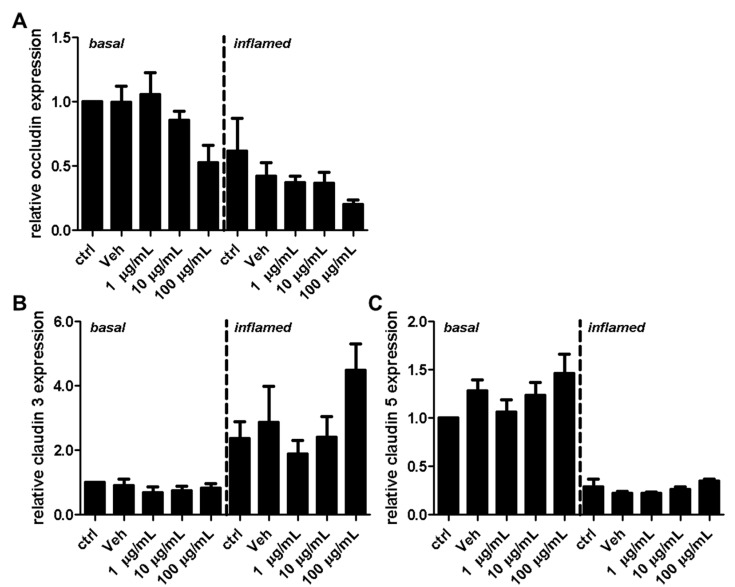
Effect of fullerenols on the relative gene expression levels of tight junctions of mouse brain microvascular endothelial cell (MBMEC) cultures under basal and inflammatory conditions. Real time PCR analysis of the expression levels of occludin (**A**), claudin 3 (**B**), and claudin 5 (**C**) in MBMEC after exposure to interferon-γ and tumor necrosis factor-α (inflamed; I + T; 100 IU each) and treatment with fullerenol (F; 1, 10 and 100 µg/mL; *n* = 3−4) for 18 h compared to cultures under basal (homeostatic milieu) conditions. *GAPDH* was used as a housekeeping gene. Ctrl, untreated control; Veh, vehicle treatment.

Next, we investigated the effect of fullerenols on the TJ proteins occludin, claudin 3, and claudin 5 through applying Western blot, immunochemistry, and gene expression analysis. Under basal conditions, fullerenol treatment (1, 10 and 100 µg/mL) did not change the amount of occludin (Figure 5A,B). Immunohistochemical analyses revealed structural alterations of occludin when fullenerol was added in the highest dose (Figure 6 row 1). When IFN-γ and TNF-α were added to MBMEC treated with 100 µg/mL, occludin was significantly decreased, whereas the two lower doses of fullerenol did not alter the amount of this protein (Figure 5A,C). Immunohistochemically, inflammatory conditions resulted in a complete structural rearangement of occludin independently of fullerenol treatment (Figure 6 row 2). When occludin was investigated on RNA levels under basal and inflammatory conditions, a trend towards a reduced gene expression of this protein was only observed for the highest fullerenol dose (Figure 7A).

Fullerenol treatment did not affect claudin 3 or claudin 5 under basal or under inflammatory conditions (Figure 5D–I; Figure 6 rows 3–6; Figure 7B,C).

### 2.3. Fullerenols Do Not Modulate the Expression of Adhesion Molecules in MBMEC

**Figure 8:**
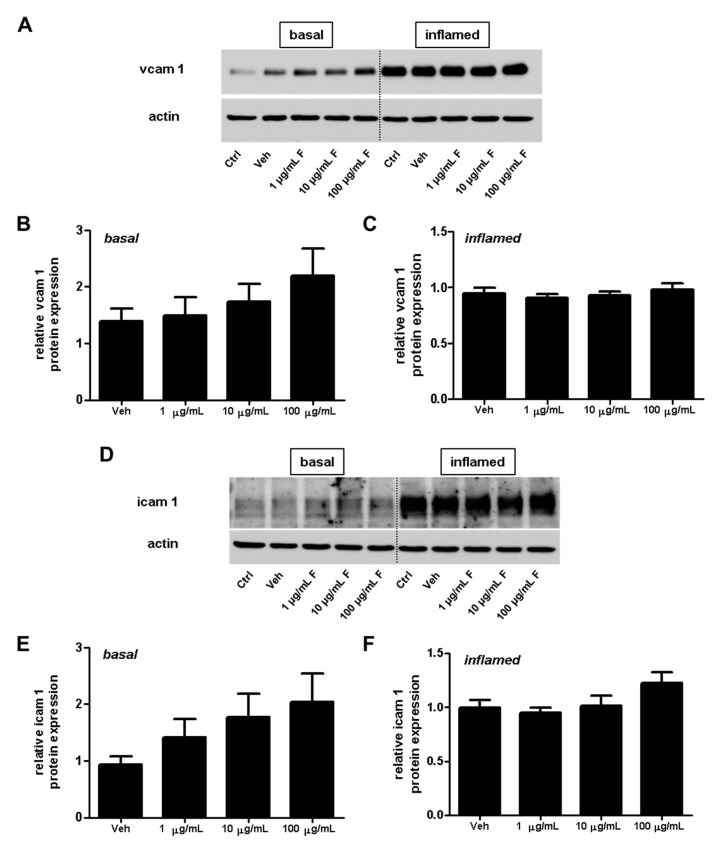
Effect of fullerenols on the amount of adhesion molecules in mouse brain microvascular endothelial cell (MBMEC) cultures under basal and inflammatory conditions. Western blot analysis and densitometric quantification (normalized to basal or inflamed untreated control cell cultures) of the amount of VCAM-1 (**A**–**C**) and ICAM-1 (**D**–**F**) proteins in MBMECs after exposure to interferon-γ and tumor necrosis factor-α (I + T; 100 IU each) (**C**,**F**), and treatment with fullerenol (F; 1, 10 and 100 µg/mL; *n* = 6) for 18 h compared to cultures under basal (homeostatic milieu) conditions (**B**,**E**). β-Actin was used as loading control. Ctrl, untreated control; Veh, vehicle treatment.

**Figure 9:**
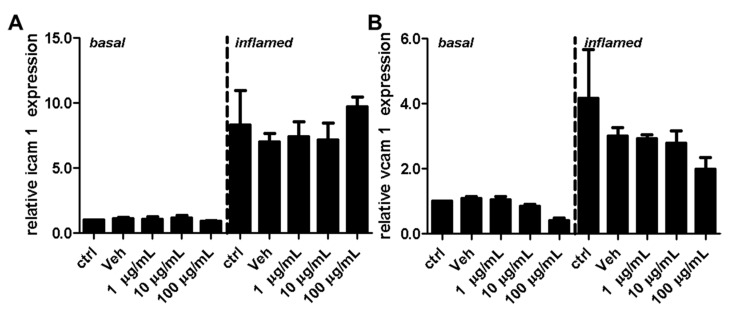
Effect of fullerenol on the relative gene expression levels of adhesion molecules in mouse brain microvascular endothelial cell (MBMEC) cultures under basal and inflammatory conditions. Real time PCR analysis of the expression levels of ICAM-1 (**A**) and VCAM-1 (**B**) in MBMEC after exposure to interferon-γ and tumor necrosis factor-α (inflamed; I + T; 100 IU each) and treatment with fullerenol (F; 1, 10 and 100 µg/mL; *n* = 4) for 18 h compared to cultures under basal (homeostatic milieu) conditions. *GAPDH* was used as a housekeeping gene. Ctrl, untreated control; Veh, vehicle treatment.

Adhesion molecules play an important role for T-cell extravasation at the inflamed BBB [24]. To examine whether fullerenols alter the VCAM-1 or ICAM-1 expression of endothelial cells, we performed protein (Figure 8) as well as gene expression (Figure 9) analysis of MBMEC, cultured for 18 h under basal or inflammatory conditions and treated with different concentrations of fullerenols. Whereas inflammation clearly increased VCAM-1 and ICAM-1 expression compared to MBMEC culture under basal conditions (Figure 8A,D and Figure 9), we detected no differences between fullerenol and control-treated MBMEC in all of the tested conditions.

### 2.4. Fullerenols Impact erk1/2 and NFĸB Activation in MBMEC

**Figure 10:**
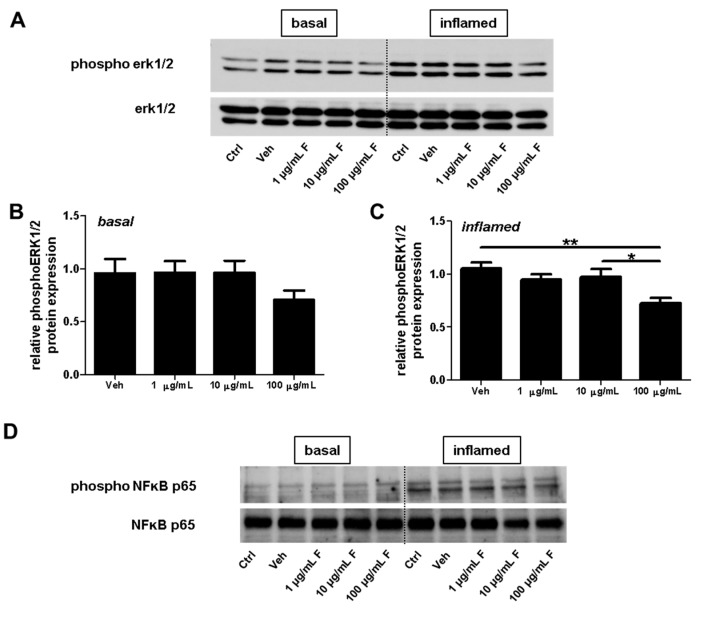

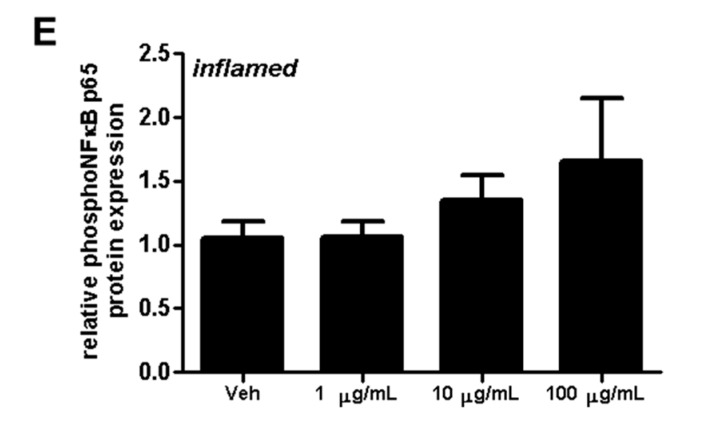
Effect of fullerenols on erk1/2 and NFκB signaling in mouse brain microvascular endothelial cell (MBMEC) cultures exposed to basal or inflammatory conditions. Western blot analyses and densitometric quantification (normalized to basal or inflamed untreated control cell cultures) of the amount of erk1/2 and phospho-erk1/2 proteins (**A**–**C**) in MBMEC treated for 60 min with fullerenols (F; 1, 10 and 100 µg/mL; *n* = 5) and exposed to interferon-γ and tumor necrosis factor-α (I + T; 100 IU each) (**C**) compared with fullerenol-treated cultures in basal milieu (**B**). Western blot analyses and densitometric quantification (normalized to basal or inflamed untreated control cell cultures) of the amount of NFκB p65 and phospho NFκB p65 proteins in MBMECs treated for 60 min with fullerenols (F; 1, 10 and 100 µg/mL; *n* = 4) and exposed to interferon-γ and tumor necrosis factor-α (I + T; 100 IU each) (**D**,**E**). Ctrl, untreated control; Veh, vehicle treatment; * *p* < 0.05; ** *p* < 0.01.

Next, the effect of fullerenol on erk1/2 phosphorylation was investigated by treating MBMEC with fullerenols under basal or inflammatory conditions (Figure 10A–C). Under inflammatory conditions, the highest fullerenol dose (100 µg/mL) significantly reduced erk1/2 phosphorylation (Figure 10C). Furthermore treatment with fullerenols dose-dependently increased inflammation-induced NFκB p65 phosphorylation (Figure 10D,E).

## 3. Discussion

Here, we investigated the effect of water-soluble hydroxylated fullerenes, i.e., fullerenols, on the BBB properties of primary brain-derived microvascular endothelial cell cultures under physiological (control/uninflamed) and inflammatory conditions. Inflammation was induced using IFN-γ and TNF-α, two cytokines, which also mediate ROS production and thus contribute to the imbalance in redox homeostasis of endothelial cells.

Changes in cellular GSH amounts can be used to determine the variability of free radicals, and thus tissue damage [25]. In the present study, the treatment of fullerenol reverted inflammation-induced reduction of GSH in MBMEC. This finding corroborates previous studies showing that fullerenol has a beneficial effect on the level of reduced glutathione e.g., in the liver [26].

As an additional finding, fullerenols at high dosages significantly reduced erk1/2 phosphorylation under inflammatory conditions. It has been reported that ROS can act as a second messenger for erk1/2 activation (phosphorylation), and antioxidants are suggested to be involved in the regeneration of erk1/2-directed phosphatase activity [9]. In this context, calcium probably plays a crucial role. Fullerenol has been shown to increase intracellular calcium concentration in endothelial cells [22]. Calcium is a signaling molecule that controls multiple processes in cells. Published evidence identified interactions between the calcium and the mitogen-activated protein kinase (MAPK) signaling pathways [27]. ERK has been the best characterized MAPK of this pathway. Therefore, our result of reduced erk1/2 phosphorylation in MBMEC treated with 100 µg/mL of fullerenol under inflammatory conditions might indirectly support the finding that fullerenol induces a G1 arrest in human umbilical vein endothelial cells (HUVEC) cultures [22], as erk1/2 signaling plays a critical role in the regulation of cell proliferation [28].

Interestingly, the expression of claudin 3 or claudin 5 did not alter under the tested conditions. Since fullerenols are potent ROS scavengers, and oxidative stress, on the other hand, significantly compromises the stringency of BBB, this observation might suggest that the integrity of tight junctions is more affected by primary inflammatory processes. This is also in line with a recently published study showing that fumarate, another agent with antioxidative properties [29], had no effect on claudin 5 expression. Furthermore, this study yielded a reduction of occludin when fullerenol was applied in its highest dose, whereas 1 and 10 µg/mL of fullerenol did not change the amount of this TJ. This result suggests further a dose-dependent effect of fullerenol on the expression of occludin. Whether ERK activation is involved in the down-regulation of occludin needs to be clarified in future studies.

Furthermore, the impact of occludin expression in MBMEC cultures on TEER remains elusive for the following reason: in our model, the TEER is already very low under basal conditions. Since the TEER is reduced to a minimum under inflammatory conditions, as expected, a reduced expression of occludin no longer decreases this parameter.

In this study, we further examined the effect of fullerenols on the adhesion molecules VCAM-1 and ICAM-1, which are responsible for a firm adhesion of T-lymphocytes, and thus enable the invasion of these inflammatory cells into the brain parenchyma. Expression of these adhesion molecules, especially VCAM-1, in cultures of inflamed cerebral endothelial cells, is regulated by NFκB activity. Collins et al. investigated the role of NFκB in the expression of cellular adhesion molecules such as VCAM-1 and ICAM-1 under inflammatory conditions [30]. The activation of NFκB is also induced by oxidative stress [31]. However, in the present study, we observed no decrease in ICAM-1 or VCAM-1 expression after treatment with fullerenol, nor a reduction in NFκB phosphorylation. In contrast, these two adhesion molecules even increased slightly when fullerenol was applied in its highest dose.

In our study, treatment with fullerenol yielded a slight but not significant increase of NFκB p65 phosphorylation. Although there are few studies revealing that NFκB contributes to cell death [32], in most cases, NFκB mediated the expression of genes, which typically promotes cellular survival [31]. It is of note that NFκB activity also influences levels of ROS by inducing the synthesis of antioxidative proteins such as glutathione [31]. Lai et al. found an increase of glutathione after the administration of fullerenol in injured tissue [16], which might be mediated by the NFκB pathway. Thus, fullerenol might further enhance the expression of NFκB, which is already induced by oxidative stress. An activation of the TLRs/MyD88/NFκB pathway by fullerene derivatives and especially an activation of NFκB have also been demonstrated by others [33,34]. Interestingly, different surface modifications of the carbon scaffold and number of carbon atoms (i.e., the size of the fullerene scaffold) appear to activate NFκB to a varying degree.

To the best of our knowledge, this study provides the first investigation of the direct effects of water-soluble fullerene on the BBB using primary MBMECs. In other endothelial cell types, such as human umbilical vein endothelial cells (HUVECs), recent reports have indicated that fullerene possess cytotoxic effects [21,22]. We also did not find differences in caspase 3 expression in fullerenol-treated MBMEC. This is in line with the results of the study by Yamawaki and Iwai [21], which demonstrated that fullerenol did not cause apoptosis. However, this finding is in contrast to the work of Gelderman et al., which displayed increased numbers of TUNEL (Terminal deoxynucleotidyl transferase dUTP nick end labeling) -positive HUVECs when treated with 100 µg/mL fullerenol [22].

We are aware of the following limitations: First, the cell culture used in this study is a monoculture system, and does not completely mimic the BBB as a 3-D model of BBB would [35]. In this study, we focused our research on MBMECs, since these cells form the innermost layer of the BBB, and thus are the cells that interact first with almost all of the components found in the blood. Second, we used IFN-γ and TNF-α in a concentration of 100 IU, which is a concentration affecting the barrier properties of MBMECs in vitro. However, in neuroinflammatory diseases such as MS, the concentration of proinflammatory agents is probably much lower. Thus, the inflamatory milieu used in this study probably only partially reflects the conditions found in vivo. Third, measured TEER values are already very low under basal conditions compared with other mammalian endothelial cell cultures, such as porcine endothelial cell cultures. When the MBMECs are inflamed, the TEER achieves an absolute minimum. Thus, we believe that a reduction of occludin expression no longer impacts TEER in this context. In conclusion, our data argue against a stabilizing effect of fullerenol on brain microvascular endothelial barrier function regarding the expression of the TJs occludin, claudin 3, and claudin 5. The expression of the adhesion molecules ICAM-1 and VCAM-1 appears to be mediated mainly by a pathway that is not modified by the biologically active properties of fullerenol. However, there is a dose-dependent effect of fullerenol on erk1/2 and NFκB activation in cerebral endothelial cells, which might counteract oxidative-induced damage of cerebral endothelial cells, and thus contribute to the protection of the neurovascular unit.

## 4. Materials and Methods

### 4.1. Materials

Fullerenol, C60(OH)_24_ was purchased from BuckyUSA (Houston, TX, USA) and dissolved in the vehicle solution composed of 0.9% NaCl. Fullerenol was diluted with cell culture medium to 1 (0.19 µM), 10 (1.9 µM) and 100 µg/mL (19 µM). IFN-γ was purchased from Miltenyi (Bergisch Gladbach, Germany). TNF-α was obtained from Peprotech (Hamburg, Germany). Mouse anti-alpha smooth muscle actin, rabbit anti-GFAP, rabbit anti-occludin and rabbit anti-VCAM-1 antibodies were acquired from Abcam (Cambridge, UK). Rabbit anti-caspase 3, cleaved caspase 3, erk1/2, phospho erk1/2 and phospho NFκB p65 were obtained from Cell Signaling (Leiden, The Netherlands). Goat anti-NFκB p65 and ICAM-1, and rabbit anti-claudin 5 were from Santa Cruz (Heidelberg, Germany). Rat anti-CD31 was obtained from Serotec (Puchheim, Germany). Rat anti-VE-cadherin was purchased from eBiosciences (Frankfurt, Germany). Rabbit anti-claudin 3 was obtained from Invitrogen (Karlsruhe, Germany). All other reagents were purchased from Sigma-Aldrich (St. Louis, MO, USA).

### 4.2. Preparation and Cultivation of MBMEC

MBMEC were isolated and cultured as described elsewhere [36]. Briefly, after removal, mouse brains were homogenized and stepwise digested by two proteases, followed by further purification steps. MBMEC were planted in 24-well plates (Nunc, Darmstadt, Germany). The cell cultures were maintained in serum-containing culture medium at 37 °C with a humidified atmosphere of 5% CO_2_/95% air, for five days. All experiments were conducted in serum-free medium.

### 4.3. Immunocytochemistry

MBMEC were cultured on collagen IV/fibronectin-coated glass coverslips. After methanol fixation for 10 min at −20 °C, cells were stained with rat anti-CD31, rabbit anti-occludin, rabbit anti-claudin 5, or rabbit anti-claudin 3, according to standard protocols. Images of MBMEC were taken with a Nikon Eclipse 50i microscope equipped with a CCD camera (Nikon, Tokyo, Japan).

### 4.4. Measurement of GSH

MBMEC were cultured in 24-well plates. GSH levels were measured using a GSH detection assay kit from Abcam, according to the manufacturer´s instructions.

### 4.5. Measurement of Transendothelial Electrical Resistance (TEER)

Electrical resistance across the MBMEC layers was measured using a resistance meter (CellZcope, San Francisco, CA, USA). TEER was assessed on endothelial cell monolayers cultured on collagen IV/fibronectin-coated Transwell Pore Polyester Membrane inserts with 0.4-μm pores (Corning, Amsterdam, The Netherlands).

### 4.6. Western Blot Assays

MBMEC were cultured in 24-well plates until they reached confluency. After cell lysis, the denatured protein was electrophoresed and transferred to a nitrocellulose membrane. Membranes were blocked for 1 h and incubated with primary antibodies at 4 °C overnight. Thereafter, the membranes were incubated with a horseradish peroxidase-conjugated immunoglobulin G antibody (Dianova, Hamburg, Germany) at room temperature for 1 h. Immunoblots were detected using ECLplus (PerkinElmer, Waltham, MA, USA) and a Kodak X-OMAT 5000 RA developer (Kodak, Rochester, NY, USA). To control protein loading, membranes were incubated with a β-actin monoclonal antibody. Bands were quantified by densitometric analysis using ImageJ Analysis Software 1.45s (National Institutes of Health, Bethesda, MD, USA) and normalized to the actin, erk1/2 or NFκB p65 band.

### 4.7. RT-PCR

RNA was isolated from MBMECs according to standard procedures. cDNA synthesis was performed using a standard protocol with random hexamer primers (Applied Biosystems, Foster City, CA, USA). Relative gene expression levels of occludin (assay ID: Mm 00500912_m1, Applied Biosystems), claudin 3 (assay ID: Mm 00515499_s1, Applied Biosystems), claudin 5 (assay ID: Mm 00727012_s1, Applied Biosystems), ICAM-1 (assay ID: Mm 00516023_m1, Applied Biosystems) and VCAM-1 (assay ID: Mm 01320970_m1, Applied Biosystems) were analyzed with a fluorescent TaqMan technology. As an endogenous control, Gapdh (TaqMan^®^ Predeveloped Assay Reagent for gene expression, part number: 4352339E, Applied Biosystems) was used. We calculated the data using the change in cycle threshold (Δ*C*_t_), ΔΔ*C*_t_, and relative quantification (2^−ΔΔ*C*t^). PCR was performed using the StepOnePlus™ Real-Time PCR System (Applied Biosystem).

### 4.8. Statistical Analysis

All results are presented as mean ± standard error of the mean (SEM). Differences between the two groups were tested with unpaired, two-tailed Student’s *t*-test. To test for significant differences between multiple groups, one-way analysis of variance was used, with post hoc Bonferroni adjustment for *p*-values. *p*-values < 0.05 were considered significant with * *p* < 0.05; ** *p* < 0.01.
